# Consumers’ opinions on warning labels on food packages: A qualitative study in Brazil

**DOI:** 10.1371/journal.pone.0218813

**Published:** 2019-06-26

**Authors:** Priscila de Morais Sato, Laís Amaral Mais, Neha Khandpur, Mariana Dimitrov Ulian, Ana Paula Bortoletto Martins, Mariana Tarricone Garcia, Carla Galvão Spinillo, Carlos Felipe Urquizar Rojas, Patrícia Constante Jaime, Fernanda Baeza Scagliusi

**Affiliations:** 1 Center for Epidemiological Studies in Health and Nutrition (NUPENS), Faculty of Public Health, University of São Paulo, São Paulo, São Paulo, Brazil; 2 Brazilian Institute for Consumer’s Defense (Idec), São Paulo, São Paulo, Brazil; 3 Department of Nutrition, Faculty of Public Health, University of São Paulo, São Paulo, São Paulo, Brazil; 4 Research Group of Digital and Information Design, Department of Design, Federal University of Paraná, Curitiba, Paraná, Brazil; University of Florida, UNITED STATES

## Abstract

This study aimed to assess consumers’ uses of and opinions on the current Brazilian food label and their reaction to the introduction of a front-of-package warning label. We conducted 12 focus groups among a diverse sample of adult consumers, to broadly assess: (1) uses of and perceptions about the current food label, and (2) opinions about implementing a front-of-package warning label to guide food purchases. Data was analyzed with a triangulation of researchers using an exploratory content analysis, which allowed codes to emerge from the data. The frequency of codes across focus groups was compared by gender and socioeconomic status to explore differences by these sociodemographic factors. Codes were divided into six main themes: (1) “Reasons for using food labels”; (2) “Barriers to using food labels”; (3) “Requirements for a new label”; (4) “Perceived influence on consumption behaviors”; (5) “Perceived influence on child behaviors”; and (6) “Perceptions of the food manufacturers using of warning labels”. Participants used food labels to check nutrient content and ingredient information but the format of these labels and the technicality of the content displayed often made the information inaccessible, particularly for those with low socioeconomic status. Most participants were supportive of the display of front-of-package warning labels on products and considered them useful to inform purchases. Women believed that they and their children would reduce the consumption of foods with front-of-package warning labels, while men reported more polarity in their intentions. For men and their children, front-of-package warning labels would result in either stopping food intake entirely or continued consumption without changes to the amount. The study results highlight the potential of front-of-package warning labels to support healthier behaviors in both consumers and their children.

## Introduction

The increase in the consumption of ultra-processed food products (UPP) plays a central role in the process of nutrition transition, defined as a shift in dietary intake and energy expenditure that coincides with epidemiological, demographic and economic changes [1]. UPP typically contain high amounts of sodium, free sugars, total and saturated fats, and trans-fatty acids [2]. Decreasing their consumption is important for safeguarding public health in Brazil, as diets high in these products have been associated with lower diet quality [3,4], and with obesity among adults and children [5].

In this context, providing nutrition information at points of purchase through food labels is likely to be a cost-effective strategy for supporting changes to a healthier dietary pattern that may protect against future non-communicable diseases [6]. This strategy is supported by international health agencies that recognize appropriate labelling of foods and drinks as a necessary environmental change for decreasing the consumption of UPP [7].

The National Health Surveillance Agency in Brazil (*Agência Nacional de Vigilância Sanitária*–Anvisa) is responsible for the regulation of food labelling, which has been mandatory since 2002. In 2003, information on calories, carbohydrates, proteins, total and saturated fats, cholesterol, fiber, calcium, iron and sodium, as well as the percentage of the daily value of each nutrient per portion was made mandatory on packaged food in Brazil. If the manufacturer includes any claim on the food package (called Complementary Nutritional Information; *Informação Nutricional Complementar*–INC), it is obligatory to declare the nutrition information of the related nutrient as well. Trans fats labelling became mandatory in 2006, when Brazil adopted the Southern Common Market (*Mercado Comum do Sul*—Mercosul) norms [8]. All nutrient information is usually presented in a nutrition facts panel at the back of the package and has to be: (1) structured in a table or presented as a text, (2) presented in culinary measurements, (3) in the country’s official language, (4) legible [9].

Some quantitative studies in Brazil with adults have indicated a low use of the current food labels by the consumers. Cassemiro *et al*. reported that only 23.0% of the 200 adults studied read the nutritional information on food labels before buying a product [10]. The study conducted by Machado *et al*. showed that, although many people might report reading the food label, most of them only consult the expiration date. In their study (n = 300), of the 81% that reported reading food labels, 91.3% consulted the expiration date while only 2% looked for nutrition information [11]. A mixed methods study by Marins *et al*. with 400 consumers in Niterói, Brazil, provided further insight into the low use of food labels—almost one fourth said they did not trust food labels and most of them could not understand the information [12].

In the current scenario of nutritional and epidemiological transition in Brazil, with increasing rates of obesity, particularly among female and lower income groups [13], it is essential to provide clear and accessible nutrition information on food labels. To target critical products such as UPP, additional measures such as the front-of-package labels (FOPL) have been proposed. These are considered particularly advantageous because of their simple message and symbol, which complements the current nutrition information displayed by the list of ingredients, the nutrition facts panel and the INC. Moreover, FOPLs have the potential to capture consumer attention, improve consumer understanding of the nutritional content of the products and encourage the use of the nutrition information [14] to make quick decisions about the healthfulness of a food product [15].

One of the proposed options for FOPL in Brazil is the warning label (WL) [16]. This model is a relatively recent format of a nutrient-specific FOPL and is displayed exclusively to indicate that key nutrients exceed recommendation levels. Interest in WLs has been growing internationally, with the model already being implemented in Chile [17] and in process of implementation in Canada [18], Peru [19], Israel [20] and Uruguay [21]. WLs aim to effectively alert the population about the presence of high amounts of critical nutrients, which can negatively affect their health [22].

There is some quantitative evidence to support the effectiveness of WLs over other options of FOPLs that display more complex information, such as the traffic-light label (TLL), which presents high and low levels of nutrients and their equivalent contribution to an adult’s daily needs. The WL had a higher impact on the food choices of 442 Chilean children, promoting the choice of healthier foods, than the TLL [23]. In Brazil, an online controlled experiment with a representative population sample (n = 3,353) showed that WLs were more effective at improving participants’ (a) understanding of excessive content of critical nutrients and (b) their ability to identify the healthier product than TLLs [24]. However, in order to better understand needs and implications of implementing WLs, it is important to complement the insights from existing quantitative work with a more in-depth assessment of the consumers’ perceptions and initial reactions to WLs.

Our study proposes to address these gaps within a diverse sample of Brazilian adult consumers. Specifically, we aim to: (1) understand their use of nutrition labels, (2) investigate the challenges faced in using these labels, (3) assess their opinions of WLs, and (4) understand how the WL might address some of the barriers of the current nutrition labels.

## Materials and methods

### Study design

We conducted a qualitative study using focus groups (FGs). Through participants’ interaction with each other, FGs can provide rich data, eliciting social norms, feelings, attitudes and perceptions about a specific topic [25]. Materials and methods of study will be reported below, following the Standards for Reporting Qualitative Research (SRQR) [26] and the Consolidated Criteria for Reporting Qualitative Research (COREQ) [27].

The current food label in Brazil was represented by the list of ingredients and the nutrition facts panel, and the WL model was represented by a black triangle with the warning “high in (name of the critical nutrient)” [28]. This model has been proposed by the Brazilian Institute for Consumer’s Defense (Idec) and developed in partnership with researchers from the Department of Design at the Federal University of Paraná, Brazil. Researchers involved in this study were nutritionists and designers, working in either public Universities in Brazil or a non-governmental institution.

### Setting

FGs were held in research facilities in four different capitals: Recife, Goiânia, São Paulo and Porto Alegre, which represent four of the five regions of the country: Northeast, Midwest, Southeast and South. The geographic distribution of the groups aimed to capture a diversity of opinion that may be influenced by regional differences in cultures, habits, contexts, and beliefs in food and nutrition.

### Sampling

Participants were invited to participate by a Brazilian research firm, thus no researcher involved in the design and analysis of this study has had any interaction with the participants. Ten to twelve people were invited to each FG, with an attendance of eight in each, totaling 96 participants. Groups were stratified by gender and socioeconomic status (SES) (high and low), with three FGs of each: high SES females, high SES males, low SES females and low SES males.

SES was assessed through the “Brazilian Criteria”, a classifying system based on the households’ possession of goods proposed by the “Research Companies Brazilian Association”. The questionnaire covers: (1) the number of bathrooms, domestic employers, automobiles, personal computers, dishwashers, refrigerators, freezers, washing machines, DVD players, microwave ovens, motorcycles, and clothes driers in the household; (2) the householder education; and (3) access to public utility services (piped water and paved street). Each response has a value from 0 to 14, depending on the item and the number of items. The sum of all responses classifies the household from A (high social class) to E (low social class) [29]. The methodology for the development of the criteria and values for each component are described elsewhere [30].

Inclusion criteria were: (a) being between 20 and 50 years of age, (b) being responsible for grocery shopping in the household, (c) shopping for groceries for the household at least once a month, (d) residing in the same city for at least three years, (e) not working or having worked for market research firms, food industries or in the health sector (nor having a family member who has), and (f) not having participated in another research discussion in the last 24 months. As reimbursement, all participants were awarded “points” that could be exchanged for a selection of giveaways. This study was approved by the Ethics Committee of the University of São Paulo (protocol n. 2.236.264) and all FGs were conducted after participants had given written informed consent.

### Procedures

The interview guide had two sections. The first section aimed to investigate participants’ opinions about the current label and identify barriers to its use. This focused on answering our two first aims (understand use of nutrition labels and challenges in using them), which was important to evaluate if the WL would be able to help overcoming the current barriers to labeling use (our fourth aim). Thus, the section included questions about information participants looked for in food labels, opinions about the clearness of food labels, barriers in reading food labels, reasons not to read the food label, among others.

In the second section, we focused on consumers’ opinions about WLs, with a focus on how they could affect consumer’s food label use. Participants saw an image of a WL and of mock-ups of different products with WLs and were asked for their opinions on implementing WLs and their expected reactions to it when shopping for food. These questions aimed to answer aims three and four: to assess opinions of WLs and evaluate if it could address some of the barriers of the current nutrition labels. A final question was used to wrap up the groups and allow for any opinions that did not emerge in the earlier discussion to be expressed. The section included questions about interpretation of the WL, reactions to seeing the WL, and the relevance of the WL.

The guide was pre-tested with a group of high SES women living in São Paulo (n = 8). After that, and because of the relevant and rich discussion that emerged about the impacts of the WL not only to the participants, but also to the children, we revised the FG guide; including one question about the predicted impact of WLs on children’s eating behaviors. The revised interview guide was used for the 12 FGs conducted from July 13^th^ 2017 to July 18^th^ 2017– three in each study location. The average duration of the FG was 2h45m, ranging from 2h39m to 3h15m. All FGs were conducted in Portuguese by a trained moderator, audio and video recorded and transcribed verbatim. Transcripts were read by PdMS and as no relevant new information was identified in the last two FGs, data saturation was considered reached.

### Data analysis

A content analysis followed Bernard, Wutich and Ryan’s recommendations, using an inductive approach to allow *a posteriori* codes to emerge from the data [31]. Thus, although codes answered the main questions in our research, new, unanticipated themes were allowed to emerge from the FG discussions. To improve the robustness of the analysis, we used a triangulation of researchers to ensure multiple views on the process of code development and application. First, PdMS extensively read all transcripts, making notes and highlighting salient aspects of the text. Exploratory coding was conducted where similar information was grouped into codes using a cutting and sorting approach. The list of codes and their meanings were discussed with FBS. After consensus was reached, PdMS developed a codebook composed of detailed descriptions, inclusion and exclusion criteria, typical and atypical examples, and an example named “close but no”, illustrating the codes’ limits.

LAM and MDU applied the codebook separately to all transcriptions using MAXQDA, version 17.0 [32]. PdMS compared both coding sheets and calculated the inter-rater reliability (IRR), the degree of agreement between coders using Cohen’s kappa coefficients, as suggested by Bernard, Wutich and Ryan [31]. The kappa is standardized and therefore can be interpreted similarly across several studies, with values above 0.8 considered almost perfect agreement [33]. Analyses were performed using GraphPad QuickCalcs (GraphPad Software, United States) (Table 1). Three codes were excluded because they: (1) showed moderate or weak IRR, as determined by kappa <0.7, (2) did not make relevant contributions to answering the research questions, and (3) were used less than 10 times by the coders. Finally, to explore differences by sociodemographic factors and to aid in the generation of future hypothesis, the frequency of codes across FGs was compared according to gender and SES of the participants. Codes are presented in the results section in bold letters and described using direct quotes (translated from Portuguese to English), paraphrases, and summary quantitative indicators.

**Table 1:** Themes and codes that emerged through content analysis of 12 focus groups in Brazil.

Theme	Code	Kappa
Reasons for using food labels	Nutrients	0.937^a^
	Ingredients	0.796^b^
	Composition	0.732^b^
	Comparison	0.817^a^
	Expiration date	0.867^a^
Barriers to using food labels	Familiarity with the product	0.746^b^
	Too small	0.851^a^
	Technical terms	0.935^a^
	Not clear	0.907^a^
	Hidden	0.808^a^
Requirements for a new label	Popular language	0.888^a^
	Clearer information	0.888^a^
	Larger letters	0.856^a^
	More visibility	0.888^a^
Perceived influence on consumption behaviors	Reducing the amount	0.947^a^
	Choose another product	0.881^a^
	Stop eating	0.856^a^
	Keep eating	0.723^b^
	Whoever wants to eat it, eats it	0.832^a^
Perceived influence on child behaviors	Children would keep eating	0.857^a^
	Children would stop eating	0.952^a^
	Learn what to eat	0.813^a^
Perceptions of the food manufacturers using of WLs	Positive perception of food manufactures	0.856^a^
	Predicted reaction of food manufactures	0.776^b^

^a^ the strength of agreement is considered ‘almost perfect’,

^b^ the strength of agreement is considered ‘substantial’.

## Results

A total of 96 participants (equally distributed across gender and SES) composed our FGs (Table 2).

**Table 2:** Division of participants in each city per gender and SES.

	Low SES		High SES	
	Men	Women	Men	Women
São Paulo	8	8	8	0
Goiânia	8	0	8	8
Porto Alegre	0	8	8	8
Recife	8	8	0	8

Codes were classified into six themes. Three concerning the first section of questions: two approaching the current Brazilian food label–“Reasons for using food labels, “Barriers to using food labels”), one about demands on how to improve it–“Requirements for a new label”. And three related to the second section of questions: two codes presented the consumers’ expected reactions to the WL–“Perceived influence on consumption behaviors”, “Perceived influence on child behaviors”–, and one code presented an unexpected discussion on the FGs about how WLs would affect consumers’ opinions about the food manufacturers–“Perceptions of the food manufacturers using of WLs” (Table 1). A final subsection entitled “Comparison of frequency of codes according to gender and SES” compares codes among groups according to gender and SES.

### Reasons for using food labels

Participants’ reported using food labels were mainly to search for the products’ nutritional characteristics through their information on **nutrients**, **ingredients** and **composition**. Expiration date was also cited. Looking for such information was important to **compare** products and decide which one to buy.

Information on **nutrients** was the most assessed among our participants. Sodium was the most searched nutrient on food labels, followed by sugar. The reason for consulting labels for this information was having a health condition that raises awareness for the need to limit the consumption of those nutrients. “In my case, I always look for the sodium information because I have a blood pressure problem” (male, low SES). Another reason was having a family member with health conditions. “I have diabetic family members, my grandmother is diabetic” (male, low SES).

Fat was also mentioned by many participants; when the type of fat was specified, participants mentioned eating less of the products with trans fat. Other less mentioned nutrients were calories, gluten and lactose, particularly when the participant or a family member was lactose intolerant. “I look if the product has lactose, stuff like that, because my son is allergic” (female, low SES). Calorie information was consulted if participants were on weight-loss diets.

Looking for information on the products’ ingredients was also frequently reported. Most participants looked for information about preservatives or food coloring, in particular when the food was targeted at children. “It [ingredients] is the first thing that I look for, because my son cannot eat anything with colorings. It’s the first thing that I look, if it [the food] has colorings” (female, low SES).

Interest in information about overall nutrient **composition** was cited less frequently. It was used to talk about the products generically, without specifying the search for a specific component. “I look at the nutrition facts panel and the product’s composition, because I saw on the TV, I don’t know if that’s true, but I saw that the first ingredients are in the highest amounts in the food” (female, high SES). This code overlapped with the codes **nutrients** and **ingredients**, as participants referred to the product’s composition when searching for nutrients’ amounts and allergenic ingredients.

As a particularity of this code, participants reported looking for **composition** information to ascertain if a product that they were used to buying was modified, especially if they noticed changes in the food packaging. “If I already know the product, I take a look at the package to see if there is anything different. If any change catches my eye, I take a better look and see if anything in the composition has changed” (female, low SES).

Apart from the products’ nutritional characteristics, **expiration date** was extensively cited, being the second information most looked for, only behind **nutrients**. Sometimes both codes, **expiration date** and **nutrients**, appearing together and reinforcing that these two information were the most important to our participants. “I look the expiration date and that sodium stuff, if I have my glasses on” (female, high SES).

Some participants consulted information on labels to **compare** products at the time of purchase, especially in terms of nutrients and price: “I have the option of a product “A” and a product “B”. I know some products, but I try to compare them, because one can be similar to another, but with a more attractive price” (male, low SES). “Light” or “whole grain” products were also compared with their regular versions to evaluate the cost-benefit of buying those products.

### Barriers to using food labels

Not reading food labels was frequently cited in the groups. The main reason was the **familiarity with the product**. The distinction made between the use of food labels among known and unknown products was evident. Some participants even said that if the recipe of a product that they use changed, they would probably not realize it, since they never pay attention to the label. In the case of known products, reading the food labels was only cited when the packaging changed. “When the package changes, we keep looking for the product that we were used to buying. Then we see that it has a new package and evaluate if it is the same thing as before” (female, low SES).

While the products not usually consumed needed to be evaluated through their label information, the known products provided a means of comparison, which was associated with the products’ brand. In addition, reading the same information on the frequently used products was considered repetitive, which also helps understanding why the expiration date was so cited by our participants. “If you always buy an orange juice, you only check its expiration date, you won’t read everything all the time, you just grab what you always buy, it is hard to change” (male, high SES).

Barriers to using food labels cited by our participants illustrate the need for time and skills to access this information, as they were hard to read (being **too small** and **hidden***)* and to understand (the information was **not clear** and usually presented in **technical terms***)*.

The most cited barrier was the letters in labels being **too small**, making it difficult to read it, especially in comparison to other information. “It’s written in very small letters all the [nutrition] information, and in the front it says “tasty” in huge letters. It makes you pay attention to the “tasty” part, not to the rest of the information” (female, high SES).

Using **technical terms** was the second most cited barrier among participants. “The sodium… the sugar… sometimes they make it too confusing. They say sucrose, saccharine… we don’t even know what that means!” (female, high SES). Some participants felt that the lack of accessibility to understanding food labels served to benefit the food industry or to meet legal requirements, but not to inform the general population. “Sometimes they make it difficult for the consumer to understand because we are uninformed in some subjects. They make it hard to understand so people won’t be inhibited to buy [the products]” (male, low SES).

Another frequent complaint was that the information was **not clear**, which could be related to its format or to the amount of information presented. “Sometimes the letters are in a dark color, the background is dark, the letters are too small, not even with a magnifying glass you can understand. It is really bad to read” (female, high SES).

Finally, having the information **hidden** was related to inconsistencies in placement.

### Requirements from a new label

Suggestions for a new food label highlighted visibility and ease of understanding of information. The most frequent demands were about understanding the labels through **popular language** and **clearer information**. According to our participants, the use of **popular language** would be more suitable for the general population, for themselves and for their family members: “Our mothers have a hard time reading [the labels], many of them don’t know these terms and cannot give meaning to them, for example, that sodium is salt. That is very worrying” (female, high SES).

**Clearer information** was suggested mostly without specification of what should be clearer. In those cases in which the participants specified what was unclear, they complained about the different terms used in the labels and the lack of organization in presenting them: “I said “clearer information” because it says “5% sugar”, and I worry about my family eating too much sugar, but in the other product it says “energy”, how much of energy is sugar? […] so, it should be clearer, organized…” (female, high SES).

Other suggestions concerned the labels’ readability, through **larger letters** or **more visibility**. Accompanied by the use of **popular language** and **clearer information**, **larger letters** would help identifying information and comparing products at time of purchase. Some participants also demanded more visibility for the information: “It could be something more highlighted, that catches your attention, that you look straight to it” (female, high SES).

### Perceived influence on consumption behaviors

When food with the WL was shown to the participants, their reactions ranged from **keep eating** to **stop eating** it. Continuing consumption, but **reducing the amount** eaten was the most cited intention: “I would not stop eating it, but I would certainly reduce it. When I like a product, I am faithful to it” (female, low SES). The intention of **reducing the amount** instead of stopping the consumption was related to how much people liked the product. The reduction was sometimes mentioned as a gradual process. Less frequently, people reported that they would eat less of other foods high in the same nutrient: “Since I am already used to using this product, I would keep buying it, but I would use with moderation other foods with the same ingredient. If it was sugar, since there is sugar in so many things, I would reduce something else” (female, low SES).

Participants also said that they would **choose another product**. The participants’ rationale to substitute products highlights the support that the WL may provide for product comparisons: “If one product has it [WL] and another doesn’t, I would buy the one without it” (female, high SES).

Participants said that they would **stop eating** the food if “it was something not important” (male, high SES) to them. This code showed a relation with time, as **stop eating** a product could either just be an initial reaction to the WL or a longer term strategy. “The day that I think that it will harm me, I will stop eating. If my blood [sugar] levels are high, I will stop it” (female, high SES).

The decision to **keep eating** was mainly related to either liking the product or being used to it: “I love condensed milk. I don’t care if it comes written in its package “if you eat this you will die”, I will keep eating it” (male, low SES).

The WL did not seem to reduce participants’ autonomy to choose products, but, on the contrary, helped them to make more conscious choices: “I think it is up to each one to decide [what to eat], I think that everything in excess is bad for you. I don’t see a problem in drinking a can of soda or one of these beverages; you just can’t do it in excess” (female, low SES). The individual’s autonomy in choosing what to eat was highlighted in the code **whoever wants to eat it, eats it**, which supported by the idea that “the decision [to eat the product or not] is yours to make” (female, high SES).

### Perceived influence on child behaviors

Opinions about children’s eating in the presence of the WL focused on either **children would keep eating** to **children would stop eating**. Among the parents believing that their **children would keep eating** food products with WLs, the main reason was the lack of interest. Less frequently, participants cited that their children would keep eating such products because of their practicality: “It is the famous emergency kit, if the food was all gone in the lunch and you don’t have more for dinner, you make an instant noodle” (male, low SES).

There was some difference in opinion between fathers and mothers in the group. While for the fathers, **children would stop eating** such foods as a consequence of an adult’s action: “I would not buy this for my son anymore” (male, high SES); mothers expected that the initiative to stop eating would come from the child. “When my daughter gets to the snack shelves and sees those warnings, I am sure she won’t want to buy them. At her school she does this. The other day we were going to buy a snack and there was a whole grain option that I didn’t know, she wanted that one” (female, high SES).

For the children to make healthier and more conscious food choices, they had to learn **what to eat**. In this sense, participants affirmed that the WL could help their children’s nutrition education, since it is accessible to children. The accessible warning could also potentially promote children’s curiosity to learn more about nutrients or healthy eating in general. This would help improving their autonomy: “From the moment that you explain to your child that this product is not good for you, he/she will start to practice [not choosing it]” (male, high SES).

### Perceptions of the food manufacturers using WL

Participants also reflected on the image of the food manufacturers that used the WL. The WL contributed to a **positive perception of food manufactures**, giving more credibility to the company. “I would start looking at that company with better eyes, because they are not only worried about selling, they are also worried with the amount of sugar” (male, low SES). The credibility was reinforced because of the Ministry of Health’s stamp. Nevertheless, when participants **predicted reaction of food manufactures**, they believed that the companies would resist adopting the WL. “I think that the person that produces will not want to use this [WL], because he will stop selling. Usually, that is how the producers’ minds work” (female, low SES).

### Comparison of frequency of codes according to gender and SES

**Fig 1:**
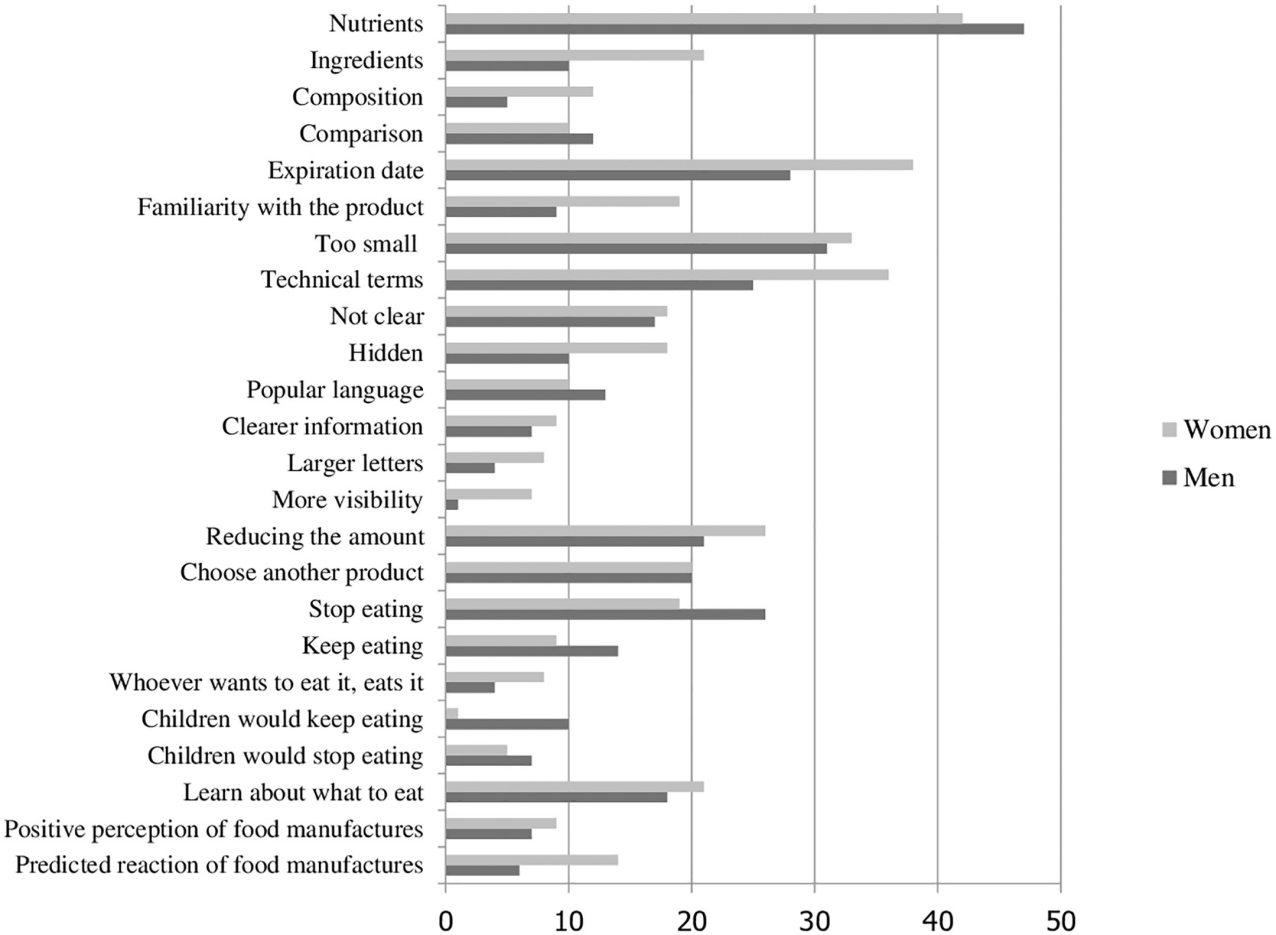
Number of times each code was mentioned, according to gender, from 12 focus groups in Brazil.

When we compared the number of times that each code was mentioned in male and female FGs, we observed a similar use of the current labels between men and women (Fig 1). Women, however, were more vocal about the barriers to using the food labels, also making suggestions for improvements, especially about the concern of presenting information in **larger letters**. When asked about their reactions to the WL, women seemed more inclined to reducing the amount of food eaten, while men tended to report more dichotomous intentions, of either stopping or continuing to eat. Differences in responses were also observed in men’s and women’s expectations of the influence of WLs on their children’s eating, with some men saying that their children would keep eating the food and women hoping that the WL would help them educate their children.

**Fig 2:**
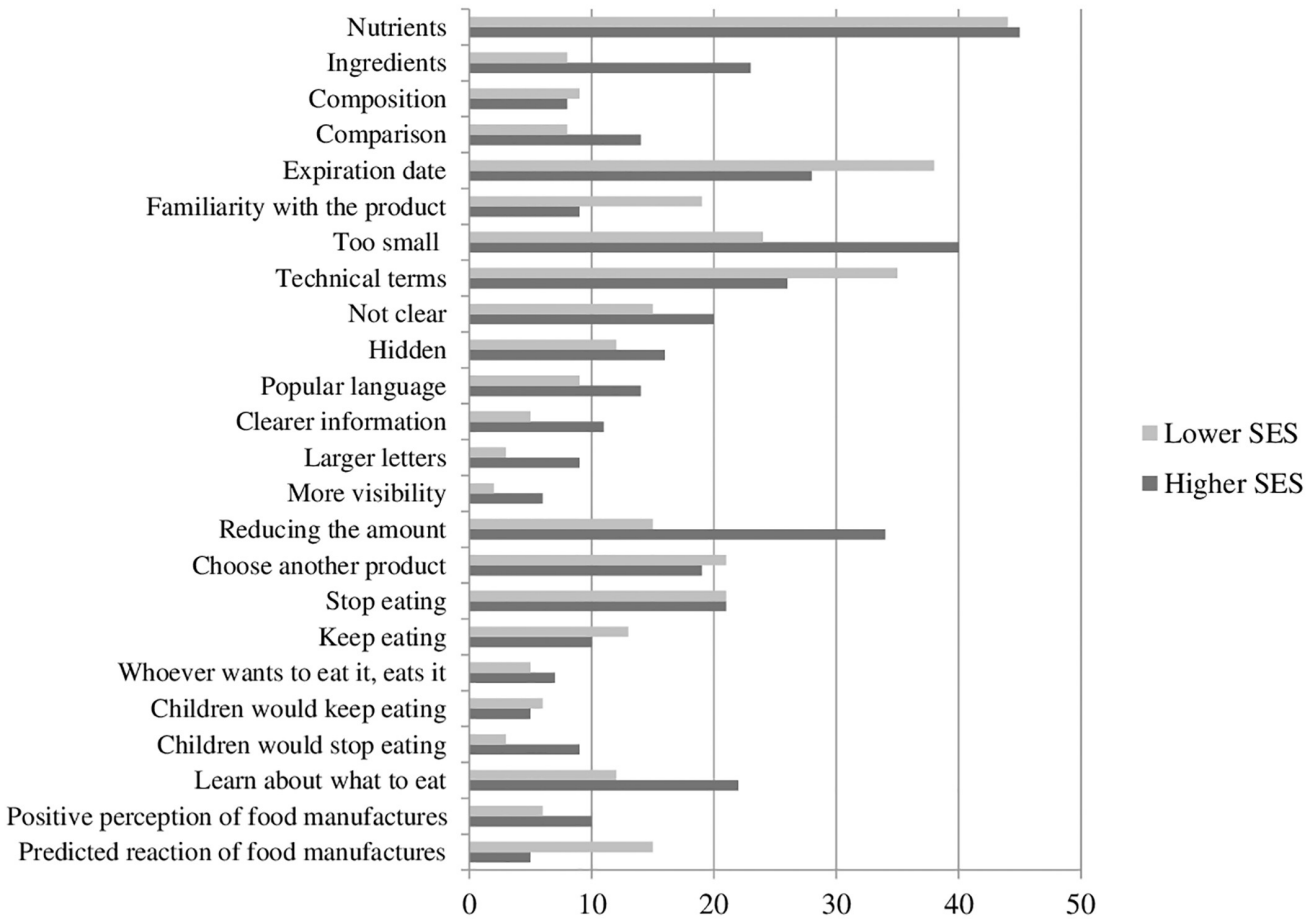
Number of times each code was mentioned, according to SES, from 12 focus groups in Brazil.

All participants looked for information about **nutrients** and **composition** in the current label, but more people with high SES mentioned using the information to compare products (Fig 2). The main barrier to the participants in the low SES was the **technical terms** used in the labels. Participants with high SES more frequently mentioned the requirements in a new food label and reacted to the WL by saying that they would **reduce the amount** of the food. Participants with low SES said more frequently that they would **choose another product** or **keep eating** the food with a WL than the ones with high SES. Differences were also observed concerning their children’s eating, as parents with high SES saw the WL as an educational tool.

## Discussion

**Fig 3:**
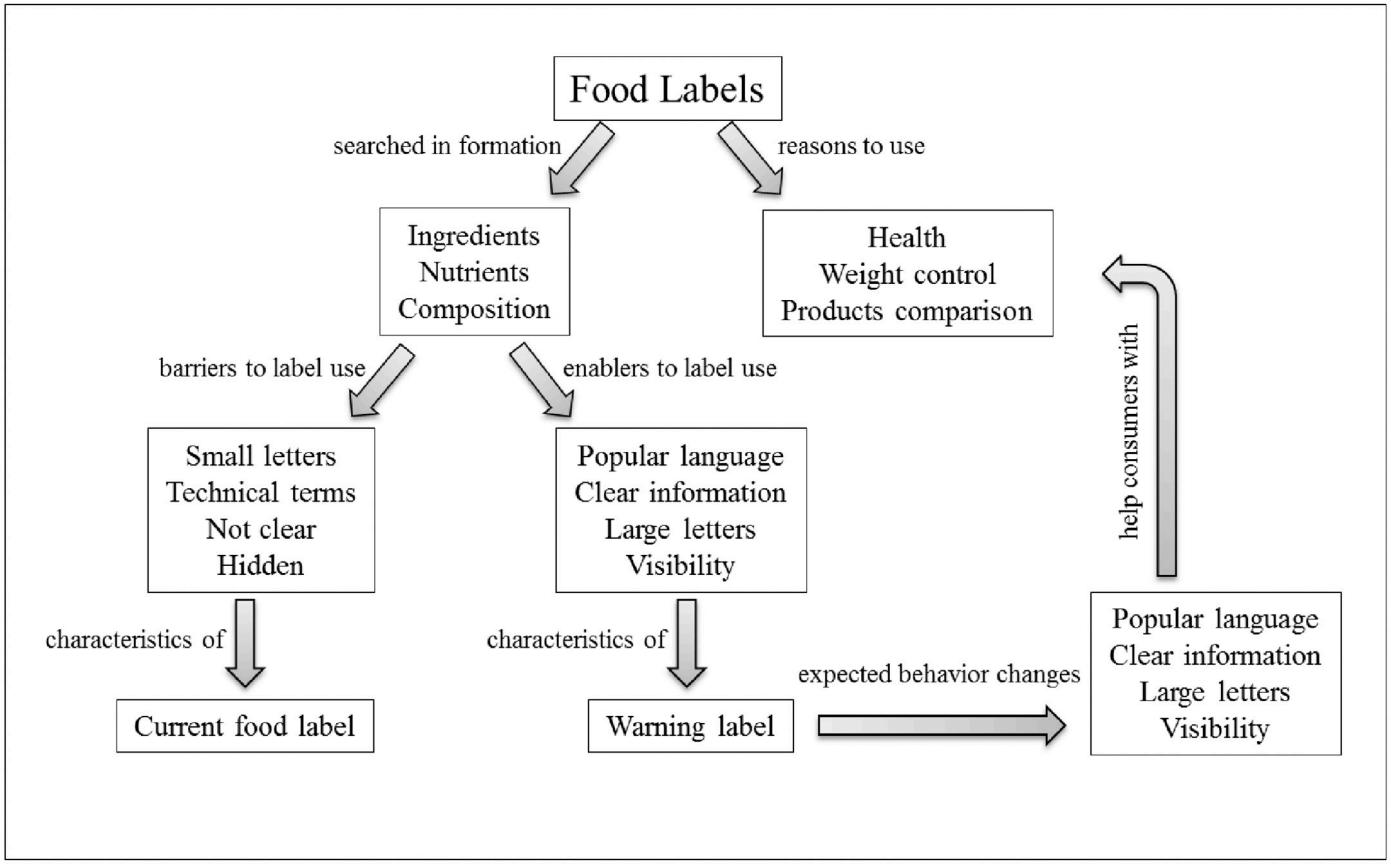
Enablers and barriers of the current label use and expected contributions of implementing a warning label.

Our results reiterate the need for accurate nutrition information on food labels and the potential of WLs to present it. Participants of the FGs were interested in assessing information that would help them make healthier food choices, in particular about the foods’ nutrients and ingredients. However, the language and format in which the current available information–i.e. list of ingredients and nutrition facts panel–is presented constitutes important barriers to its understanding and use (Fig 3).

These observations may help understand the difficulties that Brazilian consumers have in understanding food labels, as observed in the study conducted by Cavada *et al*. with 241 adults [34]. The authors described that although 48.1% of the participants read the labels of the food products; only 29.4% understood the information. Our observations also corroborate quantitative studies performed in the United States of America which described that many consumers have difficulty understanding nutrition facts panels and lists of ingredients, with the need for high health literacy to use them, in particular when they needed to perform calculations [35,36].

In our study, WLs were reviewed positively, by helping consumers identify high amounts of critical nutrients and ingredients, and compare products without needing any special skills, such as performing calculations or knowing technical terms. These benefits of the FOPL are also supported by other studies [37]. The comparison among products was also observed in the study comparing FOPLs to list of ingredients and nutrition facts panels, which showed that WLs had better results improving consumers’ ability to identify the healthier food [24]. The placement, simplicity and standardization of the information seemed to be important advantages of this model to promote consumers’ autonomy. Thus, participants could adopt diverse strategies in accordance with their priorities, such as comparing and substituting food products, decreasing consumption or reducing the consumption of other food products high in the same nutrient (Fig 3).

Our results corroborate quantitative literature that found similar trends where some consumers reported stopping purchasing the product, shifting to healthier products, or continuing to purchase the product [24,36]. In the quantitative study performed by Khandpur et al. in Brazil, the authors described an increase from 2.9% to 13% in the participants saying that would not purchase de product. In the experimental study conducted by Ares et al. in Uruguay, product substitution was the main response to WLs. Stopping purchase of the product was the response only when all food options in the category presented WLs. Among our participants, intentions of stopping to purchase and substituting were similar. However, participants also mentioned continuing to buy the same products, but in lower amounts, presenting another reaction to WLs and corroborating their potential to encourage healthier eating practices [38].

The different responses to the WL also depended on the type of product displaying it. Since the foods’ healthiness was important to the participants; the intention to consume it or not was highly related with personal taste and familiarity with that food. Understanding the interaction between taste and intention to eat is particularly relevant for UPP, as the high palatability and marketing associated to such foods make them extremely appealing to consumers, in taste and also in symbolic meaning. In this scenario, WLs can help diminish the excess consumption of UPP, through negatively influencing consumers’ appetite drive and intention to eat UPP, as observed in the controlled experimental study conducted by David *et al*. [36].

This study adds to the existing literature on food choices [39,40,41] by incorporating the role of the familiarity with the product in label reading, as participants reported not reading labels of foods they were accustomed to consuming, even though some of them recognized that the foods’ ingredients could change–which technically could mean not consuming the known product anymore–without them realizing it. In this context, consumers are more likely to notice the WL since it is displayed on the front panel and will be considered as “changes in packaging”.

In this study, the perceived safety of consuming a food product was highly related to trusting food brands, corroborating other researches that support the influence of branding on food choices [42,43]. In this sense, our results on the participants’ positive perceptions of the food manufacturers using of WLs adds to Spink *et al*.’s discussion on the value of WLs and information labels for brand marketers to enhance consumer’s confidence [44]. Thus, WLs would allow consumers to change their perceptions of not just the foods but of those who make them, the manufacturers.

Our relationship with food is dynamic and in constant construction. Therefore, we can expect reactions to WLs to change over time, as described by some participants. This observation can be compared with the study performed by Swayampakala *et al*. in Canada with smokers exposed to health WLs [45]. The authors described a decrease of attention to the WL in cigarettes overtime, but an increase of cognitive and foregoing responses. Clearly, food WLs will have the maximum effect on consumers’ behavior when newly introduced, since it will mean new packaging and new information, even on familiar products, as highlighted by our participants. However, it is important to understand how these effects translate over time–in other words: “Are changed behaviors maintained in consumers?” or “Do consumers revert to older behaviors and continue to purchase unhealthy products?” Thus, our results shed light to the importance of evaluating the effects of the WL post implementation.

It is important to also monitor the WL impacts on distinct groups, as different gender and SES showed differences in uses of the current food label and in reactions to the WL. Our results corroborate Crockett *et al*.’s argument that nutritional labelling may have different effects on distinct SES groups and contribute to understanding how food labelling may play a role in health inequalities [46]. In our study, the lower interest in nutrition information among the participants with low SES could reflect how inaccessible the current information is for this group, as illustrated by the complaint of the technical terms used in labels.

Differences were also observed between men and women, corroborating other studies that reported gender differences in food labeling use [47]. Svederberg’s observed through a qualitative study that Swedish women searched for more information on food labels than men. Our results complement the author’s discussion on gender roles and food label use. In her research, Svederberg observed that women’s concern about the family’s health reflected in their searching for more information on food labels [47]. Among our participants, the mothers’ usual responsibility for the children eating [48] reflected on their interest of how the WL could help them teach their children to choose healthier food options. The adequacy of the WLs for children is reinforced by the study conducted by Arrúa et al. that compared the influence of WLs and TLLs on 442 Uruguayan children’s snack choices. The authors described that both FOPs had a significant influence on children’s food choice, with WLs presenting higher impact than TLLs [49].

Our study has some limitations. First, we showed only the WL to the participants during the FGs, without any other kind of FOPL, which prevented comparisons. However, since quantitative studies have already focused in this comparison, we aimed to focus on the participants’ opinions on the WL that is currently being discussed in Brazil. Second, only urban, literate participants, living in key capitals of Brazil, were assessed. Thus, further studies are needed to understand the impact of WLs on rural and illiterate groups. Finally, no FG was performed in the North region of Brazil due to logistical limitations. Although the representation of all regions would be desired, we believe that capturing perceptions on four of the five Brazilian regions was enough to inform the aims of the present study. Furthermore, we did not observe important differences between regions corroborating that our FGs were enough to assess the main ideas of Brazilian consumers about food labels.

## Conclusion

This study shows a complex web of factors influencing consumers’ food label use and the expected benefits of WLs to promote healthier food choices. Although many consumers were interested in understanding information about foods’ nutrition composition and ingredients, the language and format used in current Brazilian food labels often make such information inaccessible. WLs show potential to support consumers in addressing those barriers and their suggestions for a new label. Thus, WLs seem suitable in improving consumers’ autonomy in making healthier food choices and aiding with health promotion, especially among children.

## Supporting information

S1 File Excerpts of code reducing the amount. (XLSX)
